# Key diffusion mechanisms involved in regulating bidirectional water permeation across *E. coli* outer membrane lectin

**DOI:** 10.1038/srep28157

**Published:** 2016-06-20

**Authors:** Shivangi Sachdeva, Narendar Kolimi, Sanjana Anilkumar Nair, Thenmalarchelvi Rathinavelan

**Affiliations:** 1 Department of Biotechnology, Indian Institute of Technology Hyderabad, Kandi, Telangana State 502285, India

## Abstract

Capsular polysaccharides (CPSs) are major bacterial virulent determinants that facilitate host immune evasion. *E. coli* group1 K30_CPS_ is noncovalently attached to bacterial surface by Wzi, a lectin. Intriguingly, structure based phylogenetic analysis indicates that Wzi falls into porin superfamily. Molecular dynamics (MD) simulations further shed light on dual role of Wzi as it also functions as a bidirectional passive water specific porin. Such a functional role of Wzi was not realized earlier, due to the occluded pore. While five water specific entry points distributed across extracellular & periplasmic faces regulate the water diffusion involving different mechanisms, a luminal hydrophobic plug governs water permeation across the channel. Coincidently, MD observed open state structure of “YQF” triad is seen in sugar-binding site of sodium-galactose cotransporters, implicating its involvement in K30_CPS_ surface anchorage. Importance of Loop 5 (L5) in membrane insertion is yet another highlight. Change in water diffusion pattern of periplasmic substitution mutants suggests Wzi’s role in osmoregulation by aiding in K30_CPS_ hydration, corroborating earlier functional studies. Water molecules located inside β-barrel of Wzi crystal structure further strengthens the role of Wzi in osmoregulation. Thus, interrupting water diffusion or L5 insertion may reduce bacterial virulence.

Capsules are major virulent determinants of Gram-negative bacteria involved in the evasion of host immune response^1^,^2^,^3^,^4^. They are linear or branched high molecular weight capsular polysaccharides (CPSs), typically in the order of 10^4^–10^5^ Da that are covalently or noncovalently attached to the bacterial surface^1^. With the emergence of multidrug resistance and the dearth of new antibiotics to combat bacterial infections, capsules have become a novel target for antibacterial drugs^5^. *Escherichia coli* (*E. coli*), a Gram-negative bacteria, synthesizes group1 K30_CPS_ by a Wzy polymerase dependent pathway, wherein, the tetrasaccharide repeat is biosynthesized in the cytoplasm, polymerized in the periplasm and transported to the cell exterior in a stepwise manner through a supramolecular assembly^6^,^7^. K30_CPS_ is anchored noncovalently to the bacterial surface by an outer membrane lectin, Wzi^8^,^9^,^10^,^11^. However, the surface expression and anchorage mechanisms of K30_CPS_ still remain unclear.

Here, we aim to explore the conformational dynamics of Wzi to facilitate the understanding of K30_CPS_ binding onto Wzi, an 18-stranded antiparallel β-barrel protein. Most interestingly, structure based phylogeny shows that it falls into porin superfamily. To further understand the functional role of Wzi, beyond being a lectin, we performed molecular dynamics (MD) simulations of Wzi and its mutants embedded in palmitoyloleoylphosphatidyl ethanol (POPE)/Palmitoyloleoylphosphatidyl glycerol (POPG) lipid bilayer hydrated in explicit solvent. We have shown here for the first time that Wzi can also act as a bidirectional passive water specific transporter with a diffusion permeability (p_d_) of 2.8×10^−15^ cm^3^.s^−1^, suggesting that it may play a role in osmoregulation. Strikingly, this is the first β-barreled porin found to be specific for water molecules owing to its narrow lumen.

We have identified that conserved residues at five different diffusion points of the extracellular and periplasmic faces of the barrel along with a conserved luminal hydrophobic plug play a key role in regulating Wzi water diffusion. At each entry point, water selective hydrogen bonding network regulates both water entry and exit. Three points at the extracellular side and two at the periplasmic side are found to be involved in regulating the water entry and exit through the barrel. At the periplasmic region, ‘RED’ motif acts as a major exit and entry point. At the extracellular side, while major contribution to barrel entry is through the notch region involving ‘NQQ’ motif, exit is equally through notch region and ‘YQF’ motif. Intriguingly, such a ‘YQF’ motif is observed in sodium-galactose cotransporters^12^,^13^, implicating that it may be the binding site of K30_CPS_. Thus, the dual role for ‘YQF’ triad, as a water entry point at the extracelluar side and as a K30_CPS_ anchorage point, is revealed. Results also offer stereochemical rationale for previous experimental data on the functionality/non-functionality of three H1 helix mutants^8^. This can be attributed to the observed change in the water conduction pattern or additional water entry point at the periplasmic end, corroborating the porin-like function of Wzi. As CPS is highly hydrated in nature, we envisage that Wzi may have to conduct water so as to keep the CPS in a hydrated condition. Diffusion of water molecules in and out of Wzi may be driven by the extracellular concentration of CPS.

Results also pinpoint conserved electrostatic, hydrophobic and hydrogen bonding interactions between extracellular L5 residues and the lipid bilayer which play an important role in the insertion of Wzi onto the membrane. Thus, a drug molecule that can either interfere with the water transport of Wzi or the attachment of K30_CPS_ to ‘YQF’ or with its insertion onto the membrane may aid in reducing the bacterial virulence.

## Results

### Wzi falls into porin superfamily

**Figure 1:**
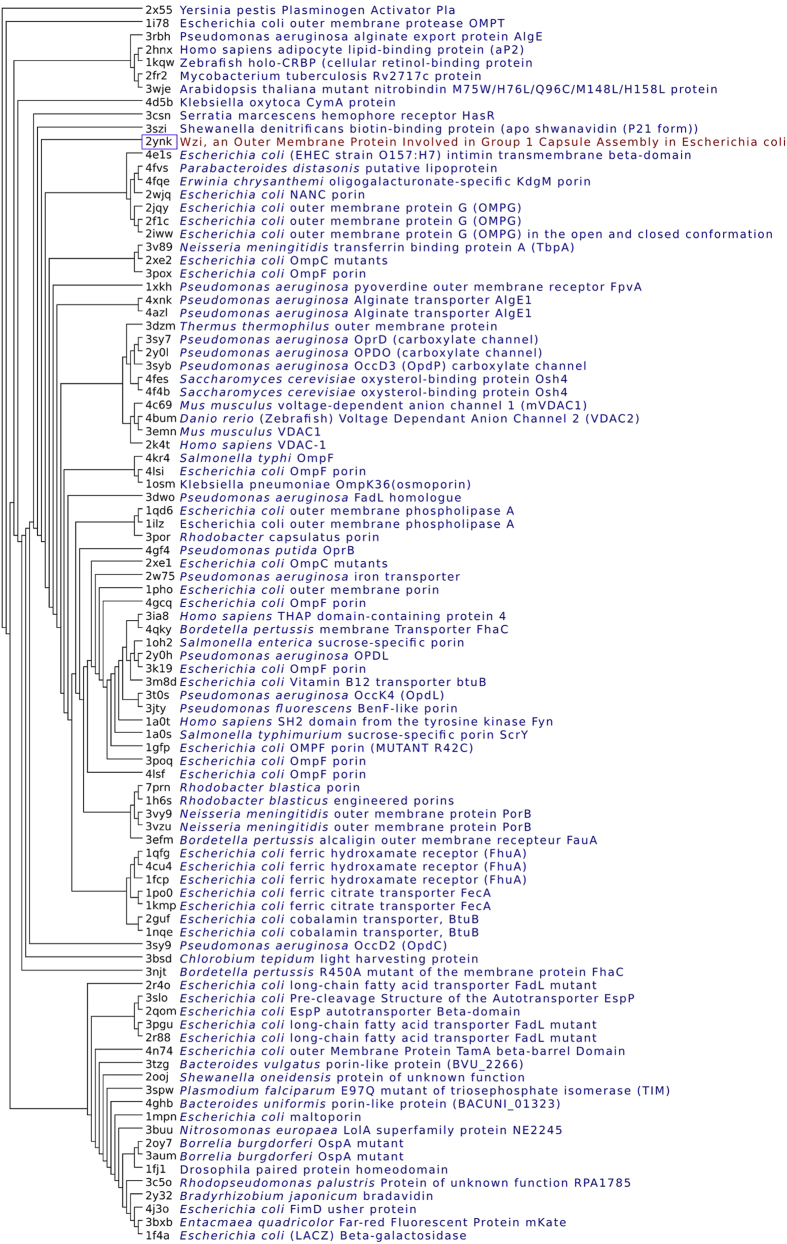
Structure based phylogenetic tree indicating Wzi as a porin. Structure based phylogenetic tree illustrating the evolutionary relationship between *E. coli* outer membrane lectin, Wzi (PDBID: 2YNK) (pink color box) with other proteins. Protein dataset was obtained from DALI by selecting proteins with Zscore>=2 when compared to Wzi. For the sake of clarity, only 92 proteins are shown in the phylogenetic tree. Wzi is boxed in pink.

As the similarity between β-barrel motif of Wzi and porins offers a clue to an additional functional role for Wzi apart from being a lectin, we have constructed a structure based phylogenetic tree (SBPT). To our surprise, results show that Wzi comes under outer membrane transporter superfamily (Fig. 1) by sharing similarity mostly with porins, in spite of high divergence at the sequence level (1–12%). In fact, such a dual function of Wzi was not realized eariler and it was emphasized to be a lectin^8^.

**Figure 2:**
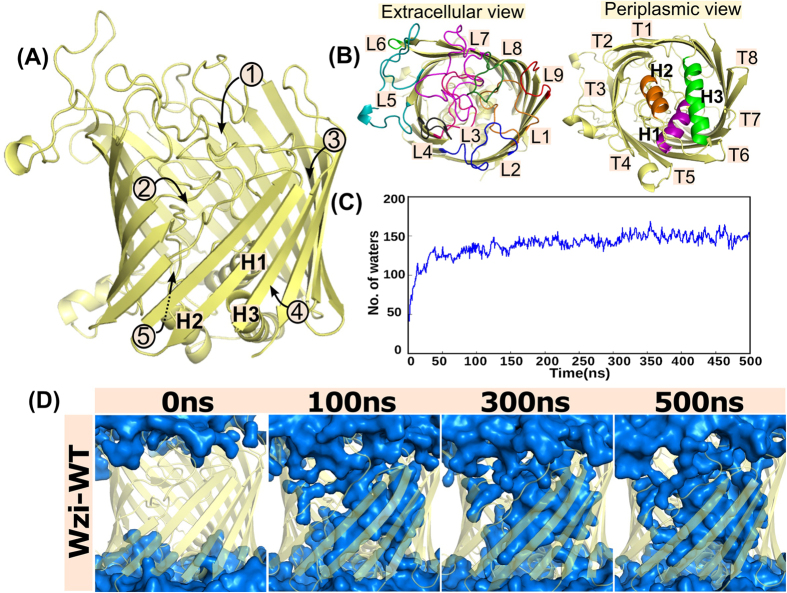
Water diffusion into *E. coli* outer membrane lectin, Wzi. Cartoon representation of 18 stranded β-barreled structure of Wzi (PDB ID: 2YNK) with the view: (**A**) perpendicular to and (**B**) along the barrel axis. In (**B**), view from the extracellular side is shown on Left, while the view from the periplasmic side is shown on Right. L1, L2, L3, L4, L5, L6, L7, L8 & L9 (Left) and T1, T2, T3, T4, T5, T6, T7 & T8 (Right) represent 9 loops and 8 turns at the extracellular side respectively and H1, H2 & H3 (Right) represent a tri-α-helical bundle at the periplasmic side. (**C**) Time vs number of barrel water molecules profile indicating ~150 molecules within the barrel at the end of the simulation. (**D**) Snapshots showing the water diffusion (blue surface) into the barrel (colored yellow) at various time intervals. Note that the barrel which is initially empty (0 ns) gradually fills up with water.

MD simulations carried out to further establish the functional role of Wzi (Fig. 2A,B) as a porin clearly indicates that water molecules gradually enter into the Wzi (herein onwards Wzi-WT) through both the periplasmic and extracellular regions and the lumen of the barrel is completely filled with the water molecules ~60 ns (Fig. 2C,D, Supplementary Movie S1). Number of water molecules within the lumen of Wzi increases steeply to ~100 over ~12 ns and gradually to ~150 during ~12–30 ns and finally stays ~150 during the rest of the simulation (Fig. 2C). Water enters into the barrel through 3 different points at the extracellular side involving conserved Y380, Q102 & F427 (marked as 1 in Fig. 2A), N153, R190, P191, N226, Q227 & Q378 (marked as 2 in Fig. 2A) and T105, Q107, Q437, W455 & T457 (marked as 3 in Fig. 2A) residues (Fig. S1).

Likewise, at the periplasmic side, water enters into the barrel through 2 different points that are located at the interspace of the β-barrel and α-helical bundle (marked as points 4 & 5 in Fig. 2A). While water entry at point 4 is regulated through H1, H3 & β-barrel encompassing residues D32, R84, R93 & E474, water flow through point 5 takes place at the interface of the turn connecting H1 & H2 and the β-barrel, involving residues D42, K301, E303, H338, H47, P215, Q247 & S244. Note that the residues involved in water passage at both the periplasmic entry points are highly conserved (Fig. S1).

### Bidirectional water permeation across Wzi

**Figure 3:**
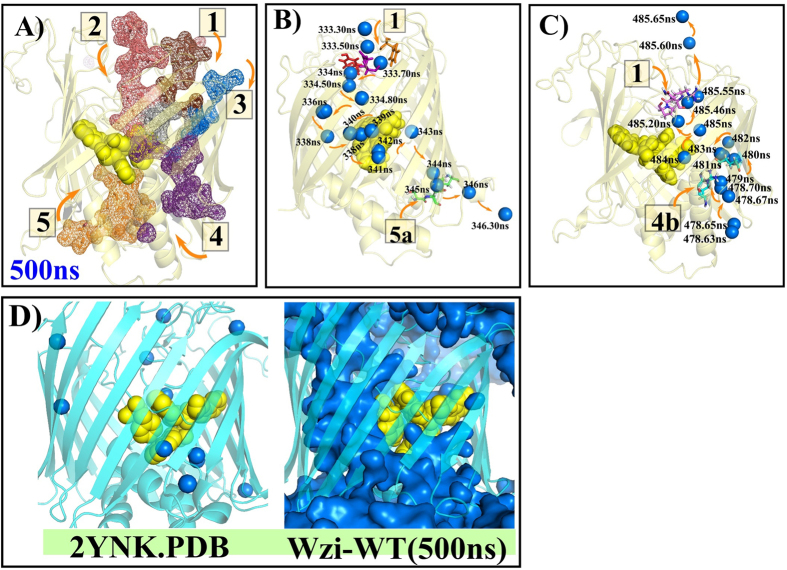
Water permeation across Wzi. (**A**) Water entry/exit (mesh representation) through points 1–5 colored brown, red, blue, purple and orange respectively. Grey region indicates the centre of the water filled lumen. Yellow spheres represent the conserved luminal hydrophobic plug. (**B,C**) Single water molecule (blue spheres) conduction pathway: from extracellular to the periplasmic side (**B**) and vice-versa (**C**) with the timescale corresponding to the water position is marked. Arrows indicate the tortuous path taken by the water molecules. (**D**) Comparison of crystal structure of Wzi (Left) (PDB ID: 2YNK) with (Right) Wzi-WT at 500 ns. Hydrophobic plug encompassing residues W53, F109, W174 and W175 is shown in yellow spheres. Note the water molecules (blue spheres) inside the β-barrel of Wzi crystal structure coincides with the water-conducting path (blue surface) of the Wzi-WT simulation. Protein is depicted as cyan cartoon and water in blue spheres.

Intriguingly, complete crossing of water molecules across the barrel, from the extracellular side to the periplasmic side (Fig. 3B, Supplementary Movie S2) and vice versa (Fig. 3C, Supplementary Movie S3), following a nonspecific pathway is also observed. Over the last 200 ns of the simulation, 17 water molecules from the periplasmic side and 21 water molecules from extracellular side cross the Wzi barrel. Such a small difference in number of water permeation events across the barrel can be attributed to the availability of a wider space on either side of the hydrophobic plug, permitting the water molecules to follow a nonspecific path. Eventually, this reflects in the time taken by the water molecules to diffuse the barrel: 8–80 ns (extracellular to periplasmic) and 6–67 ns (periplasmic to extracellular). The net flux, which is calculated just by considering the water molecules entering and exiting at the 5 water entry/exit points (See Methods for details), also remains nearly zero (0.05/ns), thus, reflecting the equilibrium state. This also reflects in the retention of total number of water molecules inside the barrel (154 ± 12/ns). Diffusion permeability (p_d_)^14^ is calculated to be 2.8 × 10^−15^ cm^3^.s^−1^.

### Mechanisms of bidirectional water diffusion into Wzi

Water diffuses into the barrel in a highly regulated manner mediated by hydrogen bonding interactions both at the extracellular and periplasmic sides through the 5 different water conduction points as detailed below.

#### Water diffusion through extracellular-entry points

**Figure 4:**
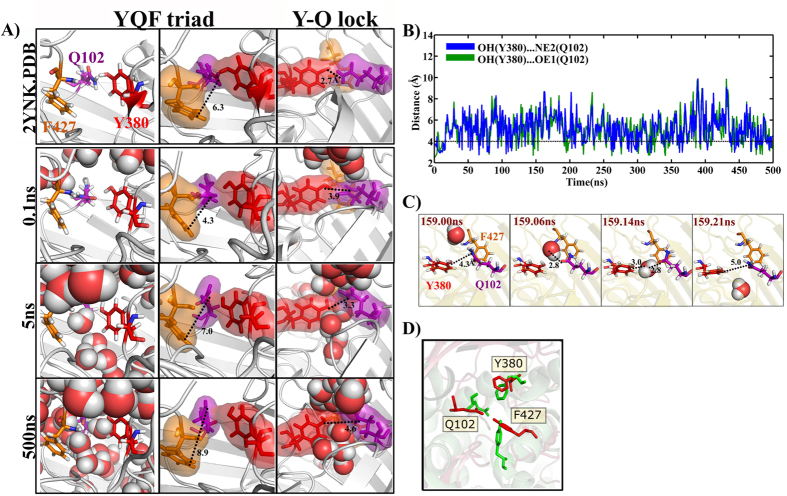
Extracellular ‘YQF’ mediated water difussion. (**A**) (Left) Snapshots showing water diffusion involving YQF triad, (Middle) distance between CZ(F427) & NE2(Q102) and (Right) Y380…Q102 lock/unlock with the corresponding simulation time marked on the left. For reference, the appropriate region in the crystal structure (2YNK) is shown on the top. Water (shown in spheres) sliding through the interface of F427 and Y380 & Q102 can be seen quite early in the simulation (5 ns, Left), during which, the water diffusion in between Y380 & Q102 is blocked due to the shorter distance between OH(Y380) & NE2(Q102)/OE1(Q102) (till ~16 ns). Note that the distance of 6.3 Å between CZ(F427) & NE2(Q102) (Middle) in the crystal structure is sufficient to diffuse water molecules. (**B**) Time vs OH(Y380)…OE1(Q102) & OH(Y380)…NE2(Q102) hydrogen bond distance profile indicating the transient opening and closing of YQ lock during the simulation after 20 ns. (**C**) Passage of a water molecule into the channel by transient opening and closing of YQ lock during the simulation is shown. (**D**) Superposition of sugar binding site of *Vibrio parahaemolyticus* sodium-galactose cotransporter (vSGLT) (PDB ID: 3DH4, colored green) and ‘YQF’ triad of Wzi illustrating their structural similarity (PDB ID: 2YNK, colored red).

Tyr…Gln hydrogen bond mediated water entry: Initial diffusion involving point 1 takes place around 5 ns through the interface between Phe(F427) and Tyr(Y380) & Gln(Q102) residues that are in hydrogen bond “locked” state (Fig. 4A). During the “locked” state, side-chains of Y380 (OH) & Q102 (NE2 and/or OE1) are engaged in hydrogen bonding interaction and stalls the water entry into the barrel till 16 ns. Subsequently, the pore gets unlocked and water enters into the channel. This can be visualized from increase in OH(Y380)…NE2(Q102) and OH(Y380)…OE1(Q102) distances to above 5 Å (Fig. 4A–C). Such locking and unlocking of the pore is observed frequently through out the simulation and regulate the water entry into the channel. YQF facilitates the entry of 23 water molecules into the barrel and permeation of 9 water molecules across the barrel during the last 200 ns. This water entry mechanism is highly conserved with the normalized frequency (NF) of 1. It is noteworthy that in the crystal structure (PDB ID: 2YNK), entry point 1 is locked by shorter OH(Y380)…NE2(Q102) (distance = 2.7 Å) and OH(Y380)…OE1(Q102) (distance = 3.9 Å) distances in contrast to the pore opened state seen during the simulation (Fig. S2). Thus, hydrogen bond between Y & Q acts as a lock for water diffusion into the barrel through YQF. Strikingly, YQF triad seen here is structurally similar to the one that is found in the sugar binding site of sodium-galactose cotransporters (Fig. 4D).

**Figure 5:**
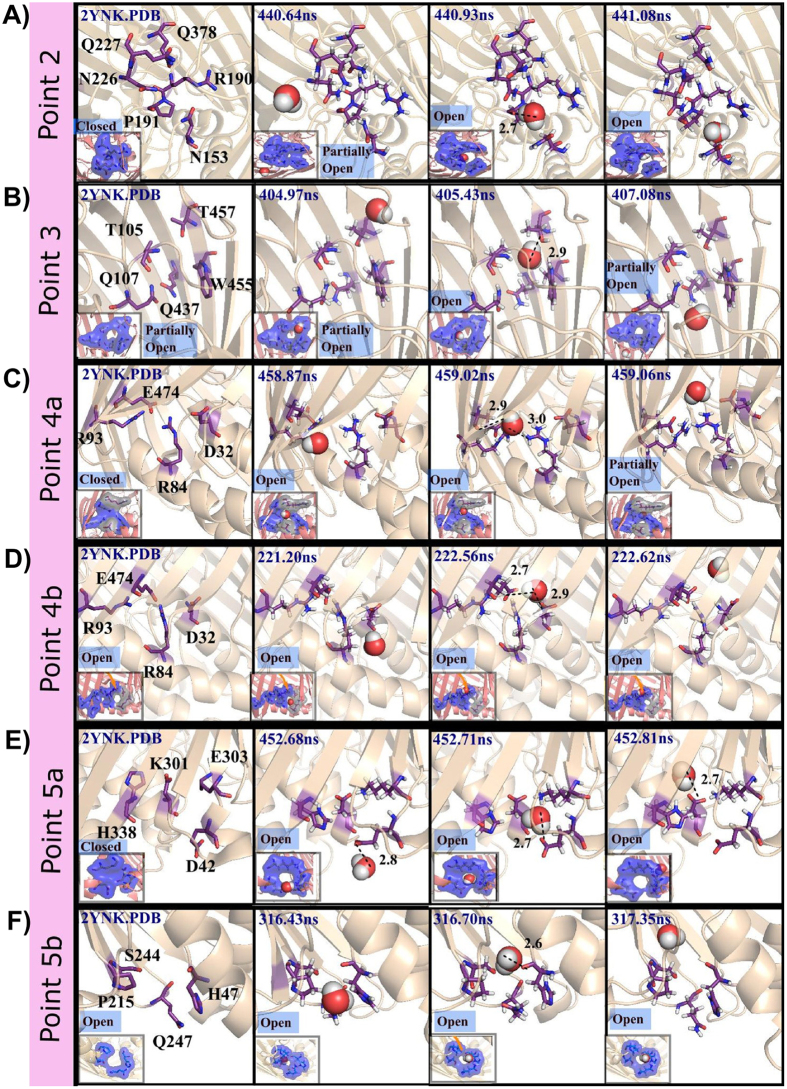
Water diffusion mechanisms. Snapshots of water entry through (**A**) point 2 involving residues N153, R190, P191, N226, Q227 & Q378 (**B**) point 3 involving residues T105, Q107, Q437, W455 & T457 (**C**) Point 4a, comprising of residues E474, R84 & R93 (**D**) Point 4b, comprising of residues E474, R84 & D32 (**E**) Point 5a involving residues D42, K301, E303 & H338 and (**F**) Point 5b comprising of residues H47, P215, S244 & Q247 along with the corresponding simulation time indicated. Periodic opening and closing of the pore to faciliate the water diffusion can be seen from the surface representation (Inset). While the residues that are involved in direct interaction with the water molecule are shown in blue surface, the ones that are not directly interacting with water molecules are shown in grey surface to just illustrate the pore formation. For reference, the crystal structure of Wzi is shown in the left.

Water diffusion through Asn…Gln hydrogen bond network: Water entry via the notch is regulated by the pore formed by the extracellular residues, Asn(N153), Arg(R190), Pro(P191), Asn(N226), Gln(Q227) & Gln(Q378) that is in closed state (point 2, Fig. 5A). As a water molecule approaches the notch region, it hydrogen bonds primarily with N226/Q227/Q378 (‘NQQ’ motif) by forming a pore and further diffuses into the channel involving N153, R190 & P191. After the water moves into the barrel, the pore again enters into the closed state as found in the crystal structure and can be visualized in Fig. 5A. Thus, the pore opening and closing involve side chain rearrangement of the aforementioned residues and facilitate water diffusion through point 2. Such water entry mechanism is highly conserved (NF = 0.87). Through this mechanism, 84 water molecules enter into the barrel that is ~3.5 times higher compared to entry point 1 and contributes to the diffusion of 12 water molecules to the periplasmic side.

Threonine flipping: At the entry point 3, water diffusion happens through hydroxyl group flipping (NF = 0.92) of T457 (Fig. 5B). The mechanism is such that, when a water molecule enters into the channel, the hydroxyl group of T457 side chain hydrogen bonds with the water molecule and pushes it inside the channel by flipping. It then breaks the hydrogen bond with T457 and slides into the channel through aromatic W455. Other amino acids that facilitate the consequent movement of water inside the barrel are T105 & Q107. Entry point 3 participates in entry of 11 water molecules into the barrel that is significantly lower compared to entry point 2 (~9 times).

#### Role of charged amino acids in periplasmic water entry

Besides the water diffusion into the barrel from the extracellular side, water also diffuses from the periplasmic side of barrel during the simulation through 2 different entry points.

Regulation of water diffusion through bifurcated salt bridge: At the entry point 4a of the periplasmic side, water regulation is mediated by R93 & E474 of the β-barrel and R84 of H3 that together form bifurcated salt bridge. When a water molecule appears in the vicinity of these residues, it hydrogen bonds alternately with the amino groups of R84 & R93 and carbonyl group of E474 and invades slowly into the barrel without disrupting R93 & E474 and R84 & E474 salt bridges (NF = 1) (Fig. 5C). A second entry point (denoted as 4b) located adjacent to R84 & E474, involves D32 of H1 (‘RED’ motif) that forms a hydrogen bond with the water molecule and pushes it inside the barrel (NF = 1) (Fig. 5D). Entry points 4a and 4b together contribute to water entry through point 4. However, water entering through point 4b is predominant (50 water molecules) compared to 4a (4 water molecules). Entry point 4 transports 17 water molecules to extracellular side.

Water entry through negatively charged periplasmic residues D42 & E303: Water entry through point 5a at the periplasmic side (Fig. 5E) is initiated by the attachment of water oxygen to carboxylic group of Glu303, a barrel residue, through hydrogen bonding interaction. Upon discharging from this hydrogen bond, it moves into the barrel by networking with Asp42 (helix 1), Lys301 (barrel) and H338 (barrel). This mechanism is highly conserved with NF = 1. Number of water molecules enter through point 5a (15 molecules) is significantly lower than the point 4b (50 molecules).

Water entry through H47-P215-Q247 triad: Point 5b at the periplasmic side favors only 2 water entries into the barrel (Fig. 5F) through a pore encompassing the barrel loop residues H47-P215-Q247 (Fig. 5F). This is initiated by a hydrogen bond formation between the water molecule and the side chain amino/carbonyl group of Gln247. Subsequently, the water moves inside the barrel by establishing hydrogen bond with backbone carbonyl group of H47 and OH of S244 (NF=1).

#### Barrel water exit mechanisms

Extracellular water exit: Not surprisingly, mode of water exit at the extracellular side involves same residues as the water entry (Fig. S3). At the extracellular side, both points 1 & 2 act as the major outlet by promoting the exit of 46 & 41 water molecules respectively. This is significantly higher compared to point 3 that exits only 6 water molecules.

Periplasmic water exit: Among the periplasmic entry points 4a & 4b, the latter promotes (32 water molecules) more water exit compared to the former (7 water molecules). Points 5a & 5b contribute more towards water exit (31 & 18 molecules respectively) compared to the entry (15 & 2 molecules respectively).

Eventually, the extracellular and periplasmic residues that are involved in water entry are involved in exit also (Fig. S3).

### Formation of transient water diffusion wires

Development of transient water wires (Fig. S4) associated with aforementioned water diffusions (both entry & exit) are observed at each diffusion point (Fig. 3A). As the water entry & exit happens at random time intervals due to the varying residence time of the water molecules inside the barrel, both of them simultaneously contribute to the formation of the water wires. Breakage in these transient water wires is observed based on the entry & exit of the water molecules. A single water wire is observed in all the diffusion points except at the extracellular point 1, wherein, it accommodates 2 transient wires.

### Lumen hydrophobic plug plays a key role in regulating Wzi water conduction

A hydrophobic plug present at the barrel center and protruding towards the lumen, governs the water transport across the channel. The plug encompasses conserved aromatic residues (Fig. S1) W174 & W175 of L3, F109 of L1 and W53 that is part of the connecting loop between periplasmic α-helices H1&H2 along with nonaromatic hydrophobic residues (Ala, Val, Leu and Ile). It eventually narrows down the channel at the center and confines the movement of water molecules inside the channel through a specific path. This can be clearly seen in Fig. 3A, wherein, the water molecules entering from multiple diffusion points from the periplasmic (2 diffusion points) and extracellular (3 diffusion points) sides merge at the region in between the hydrophobic plug and the β-barrel. Thus, the hydrophobic plug acts as a junction for the water molecules entering from both the sides.

### Pentaglycine mutants at the periplasmic end of helix H1 alter water diffusion pattern

**Figure 6:**
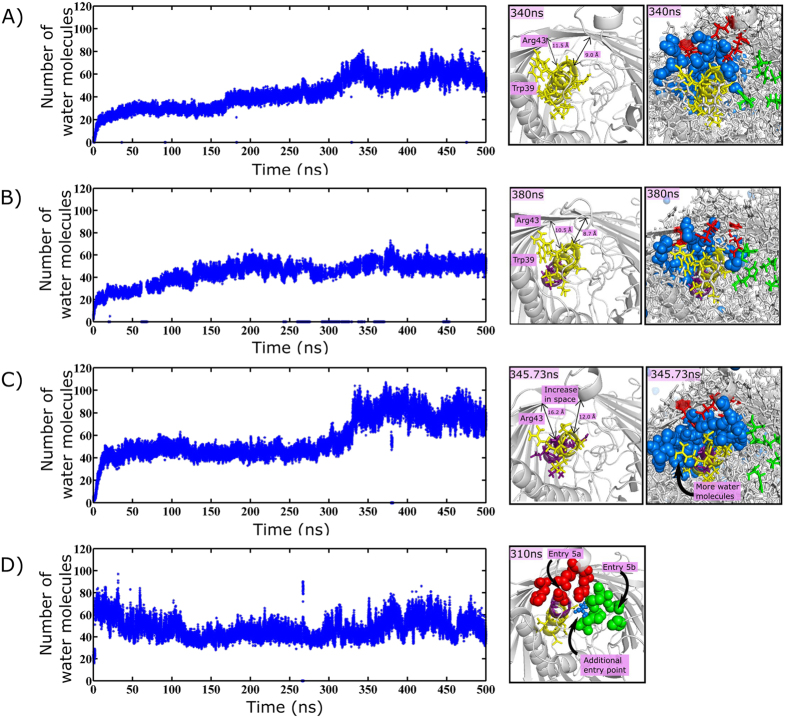
Comparison of water diffusion patterns in Wzi-WT and three helix H1 mutants. Time vs number of water molecules at the interface of Helix H1-beta barrel corresponding to Wzi-WT ((**A**), Left), H1mut1 ((**B**), Left), H1mut2 ((**C**), Left) & H1mut3 ((**D**), Left). The corresponding cartoon representation (grey) at the periplasmic end is shown in the Right. Note the increase in number of water molecules (~80) at the interface of H1mut2 associated with the increase in the interspace ((**C**), Right) compared to Wzi-WT ((**A**), Right) and H1mut1 ((**B**), Right) (~60). Additional water entry point observed in H1Mut3 ((**D**), Right) during the closed state of entry 5b (colored green) can be seen. Note that such additional entry point is not observed in Wzi-WT & H1mut1. W39 (shown in stick) that acts as a blocker in Wzi-WT and H1mut1 is absent in H1mut2. R43, along with W39 (shown in stick) at the periplasmic end is absent in H1mut3. These lead to the difference in water conduction pattern of H1mut2 and additional entry point in H1mut3, thus, may attribute to their deleterious nature. H1 residues are shown in yellow and pentaglycine mutants are colored as purple.

To elucidate the reason behind experimentally observed difference in the functional/nonfunctional forms of pentaglycine helix H1 mutants^8^, viz., H1mut1 (residues 32–36 mutated to Gly), H1mut2 (residues 35–39 mutated to Gly) and H1mut3 (residues 39–43 mutated to Gly), MD simulations have been carried out for these mutants. Strikingly, water diffusion patterns of H1mut1, H1mut2 & H1mut3 are quite different from that of Wzi-WT (Fig. 6). In fact, the number of water molecules at the interface of helix H1 and the beta barrel is significantly altered between different mutants, due to the absence of longer side chains in H1mut2 (W39) and H1mut3 (W39 & R42) (Fig. 6, Left). While the nondeleterious H1mut1 is able to retain the number of water molecules at the H1-barrel interface same as that of Wzi-WT, H1mut2 & H1mut3 exhibit significant difference. This is due to the fact that the pentaglycine mutation is located inside the barrel for H1mut1 (Fig. 6B), while it is located at the periplasmic surface for H1mut2 (Fig. 6C) & H1mut3 (Fig. 6D). In H1mut2, significant movement in H1 is observed and as the result, it accommodates more water molecules at the H1-barrel interface. Thus, the water diffusion pattern of H1mut2 is different from Wzi-WT (Fig. 6C). In H1mut3, as one of the glycine mutation overlaps with the entry point 5b residue (Asp42), the water diffusion pattern is altered (Fig. 6D). Interestingly, there is an additional entry point seen at the interface of H1 and entry point 5b, when 5b enters into the closed state. Such additional entry point is not observed in Wzi-WT. Thus, it is clear that H1mut2 & H1mut3 have different diffusion patterns compared to the Wzi-WT. These differences may significantly affect water conduction across the channel under osmotic stress. This may be the reason for their deleterious nature in sharp contrast to the nondeleterious H1mut1 that maintains similar diffusion characteristics as that of Wzi-WT.

It is noteworthy that all the above pentaglycine mutants retain their α-helical conformation (Fig. S5). This is in line with an earlier study, which shows that tri- and hexa- glycine can adopt α-helical conformation^15^.

### Ion binding pocket at the extracellular side

**Figure 7:**
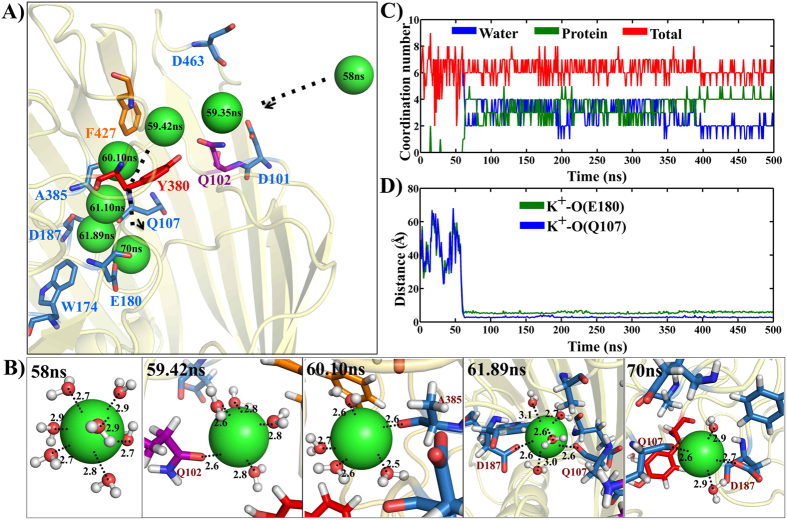
Extracellular ion binding pocket of Wzi. (**A**) Entry of a single potassium ion (green spheres) from the extracellular side into the ion pocket through ‘YQF’ triad with the position specific time marked. The amino acids with which the ion finally harbours are shown in stick representation (colored blue) with carbonyl oxygen colored yellow. W174 that forms part of the hydrophobic plug is indicated in blue colored stick. (**B**) Snapshots at various simulation times (marked) indicating the coordination of K^+^ (green spheres) with water (ball and stick with water oxygens colored red) and protein. (**C**) Time vs ion coordination number profile calculated using a cutoff distance of 3.6 Å^48^. Overall (red) and decomposed (water (blue) & protein (green)) ion coordination numbers are shown. (**D**) Distance between potassium ion and the carbonyl oxygen of E180 (green) and Q107 (blue) plotted over 500 ns simulations.

Another important observation is the tight association of a positively charged potassium ion onto the extracellular surface of Wzi. The ion starts entering into lumen ~58 ns by coordinating with the side chain carbonyl group of Q102 and through subsequent conjoint coordination with the hydroxyl group of Y380. It further moves inside the channel at the interspace between F427 & Y380…Q102 of ‘YQF’ triad (Fig. 7A) coinciding with water diffusion point 1 (Fig. 4). The ion interacts with the aromatic side chains of Y380 & F427 through coordination bond and cation…pi interactions respectively and subsequently, coordinating with the carbonyl oxygens of Q107, E180, D187 and A385 in an alternating fashion (Fig. 7A,B). The water shell surrounding the ion in bulk solution is partially stripped off as it enters the protein (Fig. 7B) and the dehydration energy is compensated by interaction with carbonyl oxygens of the ion binding pocket residues. Thus, the ion maintains an average coordination number of ~7 (Fig. 7C), coordinating with water molecules alone at the beginning and then with both water and protein as it enters into the pocket. A shorter distance of ~5 Å between the ion and the backbone carbonyl oxygens of E180 and Q107 beyond ~60 ns indicates the strong association of the ion with the binding pocket (Fig. 7D).

### Role of extracellular loop L5 in membrane insertion

**Figure 8:**
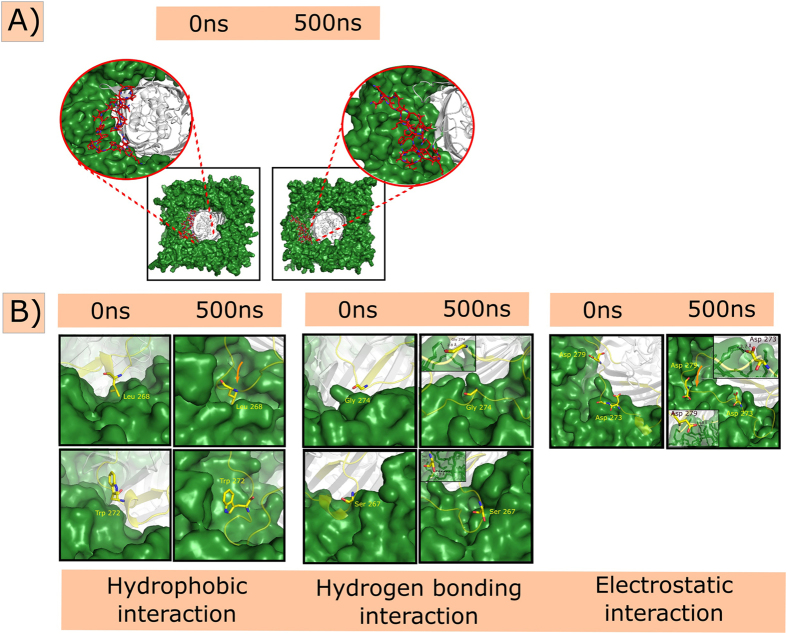
Role of extracellular loop 5 (L5) in membrane insertion. (**A**) Snapshots depicting the splaying of L5 (colored red) and its insertion onto the membrane (green surface) during the simulation. Wzi is shown as grey colored cartoon. L5 is zoomed to illustrate the splaying of L5 onto the membrane. (**B**) Snapshots at 0 ns and 500 ns illustrating crucial interactions between L5 and POPE/POPG membrane facilitating Wzi anchorage: (Left) hydrophobic interactions involving L268 & W272, (Middle) hydrogen bonding interactions with G274 & S267 and (Right) electrostatic interactions with D273 & D279 are shown. Zoomed view of interaction at 500 ns is shown as Inset.

Wzi-WT simulation indicates that L5 is not involved in the water conduction pathway. However, it plays a major role in membrane insertion. Though the L5 residues are initially aligned close to the barrel and are not involved in any interaction with the membrane (Figs 2B and 8A), they slowly splay out during the simulation by undergoing large conformational changes and insert into the membrane ~50 ns (Fig. 8A and Movie S4). This is facilitated by complete burial of residues 266–276 onto the membrane surface. One among the key interactions involved in the anchorage of the protein into the membrane is the hydrophobic interaction mediated by highly conserved W259, E266, F271&W272 (Fig. 8B) and semiconserved L268 (Fig. 8B & S1) residues. It is noteworthy that L268 is replaced by F268 in some species, which can also participate in such hydrophobic burial. Another important interaction that facilitates membrane insertion is the electrostatic interaction between the positively charged amino head group of POPE and negatively charged carboxylic group of conserved D273&D279 (Fig. 8B (Inset)). Hydrogen bonding interaction between side chain hydroxyl group of conserved S267 & phosphate oxygen of POPE and the head group of POPE & carbonyl group of G274 also participate in this anchorage.

## Discussion

We have shown here for the first time through structure based phylogeny and MD simulations that *E. coli* outer membrane lectin, Wzi also functions as bidirectional water selective porin, offering justification for its β-barrel fold. Note that although Wzi is specific for water conduction like aquaporins, both are structurally very different, as the latter possesses an alpha helical structure.

Five water diffusion points are identified (two at the periplasmic and three at the extracellular sides), through which, the water entry/exit from extracellular & periplasmic sides occur in a highly regulated manner in Wzi (Figs 3, 4, 5, S3 & S4). These mechanisms aid in retaining the total number of water molecules inside the barrel ~150 and facilitate complete crossing of water molecules across the channel by passive diffusion. Nonetheless, water-crossing time varies even through the same diffusion point, as the water molecules don’t take up a specific transportation pathway due to the wider lumen. Diffusion permeability of Wzi is estimated to be 2.8×10^−15^ cm^3^.s^−1^ that is 2 times lower than that of aquaporin monomers as reported in previous MD simulations^14^,^16^.

A novel ‘YQF’ triad motif located in the extracellular side is identified to be one of the major diffusion points (Fig. 4). Notably, a similar triad is found in the water and sugar entry path of sodium-galactose cotransporter (SGLT) (PDB ID: 3DH4^17^) that is highly conserved among several bacterial sugar transporters (Fig. S6). Thus, we propose that ‘YQF’ triad may be the K30_CPS_ binding motif. Interestingly, ‘YQF’ triad also participates in potassium ion conduction into the ion-binding pocket at the extracellular side (Fig. 7). This further implicates that it may be the anchoring point for the negatively charged K30_CPS_ onto Wzi.

A hydrophobic plug that is constituted by conserved W53, F109, W174 & W175 residues (Fig. 3) and located inside the channel acts as a physical barrier for water molecules to permeate. Consequently, transient water wires formed at multiple entry points merge at the space between the hydrophobic plug and the barrel (Fig. 3A and S4). Thus, the hydropbhobic plug acts as a junction for water molecules from multiple entries and redirects them to take a specific conduction path. Such water permeation and regulation across Wzi are also confirmed by preliminary 50 ns MD simulation carried out using CHARMM molecular modeling program^18^,^19^ (Fig. S7).

MD simulations carried out on three pentaglycine substitution mutants of helix H1 indicate that H1mut2 & H1mut3 exhibit different diffusion patterns in contrast to H1mut1 & Wzi-WT (Fig. 6, Table S1). This is due to the fact that conserved W39 and/or R43 residues that act as a blocker in Wzi-WT & H1mut1 are absent in H1mut2 (W39) & H1mut3 (W39 & R42) and lead to difference in water diffusion. While H1mut2 accommodates more water molecule at the H1-barrel interface, H1mut3 favors an additional entry point, resulting in different water diffusion pattern compared to Wzi-WT. Incidentally, H1mut2 & H1mut3 mutants are shown to be experimentally nonfunctional^10^ implicating that any alternation in barrel diffusion may significantly alter the function of Wzi. In fact, this offers an indirect evidence for the water conducting property of Wzi.

SBPT along with water molecules inside the crystal structure of Wzi β-barrel further supports the water transporting property of Wzi (Fig. 1) and hence, its role in osmoregulation. Thus, molecular dynamics simulation along with structure based phylogeny establishes the water transporting function of Wzi. Interestingly, among the 322 non-redundant proteins that exhibit strucutral similarity with Wzi, none are found to be lectins (majority of them are bacterial porins). This indicates that the protein might have evolved the lectin function when the bacteria established the self-defense mechanism for its survival.

Based on the bidirectional water permeation property of Wzi, we propose here a mechanism by which it may act as an osmosensor. Under hyperosmotic condition, wherein, the K30_CPS_ that is more on the bacterial surface may exert high osmotic pressure and hence, Wzi may transports water from periplasmic to the extracellular side to avoid the rupturing of the cell. This may further aid in keeping the K30_CPS_ in a hydrated condition as well as prevent the accumulation of nascent K30_CPS_ onto the bacterial surface. On the other hand, under hypoosmotic condition (less concentration of K30_CPS_), water molecules move from extracellular to periplasmic side to maintain the osmotic pressure. Thus, water transport across Wzi depends on the concentration of K30_CPS_ on the bacterial surface. Inferred association between additional water diffusion pattern and deleterious nature of periplasmic helix mutants H1mut2 & H1mut3 is in supportive of the role of Wzi in osmoregulation. In fact, relationship between capsular polysaccharide production and osmotic pressure is an already existing fact^20^,^21^,^22^. This study also proposes that ‘YQF’ triad acts as the binding site for K30_CPS_ onto Wzi and pinpoints the importance of extracellular L5 in the anchorage of Wzi onto the outer membrane. Thus, disrupting the interaction between K30_CPS_ & ‘YQF’ triad or altering the Wzi water diffusion or disrupting the interaction between L5 & membrane may be attractive strategies to reduce the bacterial virulence.

Besides establishing the role of Wzi as a porin, this study further establishes the importance of molecular dynamics and structure based phylogeny in identifying the unknown function of a protein. Thus, these techniques together can be used to identify the function of hypothetical proteins as well as understand their functional evolution.

### Experimental procedures

#### Structure based alignment

As Wzi is highly divergent with respect to other family of porins (midnight zone with less than 12% of homology) at the sequence level, structure based alignment is performed to illustrate the role of Wzi as a porin. Using DALI^23^ structure based alignment server, 532 structures that have a Z-score greater than or equal to 2 with respect to Wzi are obtained. After removing repeated chain id’s, a net total of 328 unique pdb structures are procured. This is further reduced to 322 after removal of obsolete pdb entries. Subsequently, a diagonal matrix encompassing the RMSD based quality function that describes the Ca alignment (Q-score) is developed^24^ using in-house programming. Note that the RMSD used in the estimation of Q-score between the Ca atoms of any two proteins in the matrix is calculated using Pymol software (The PyMol Molecular Graphics System, Version 1.3, Schrodinger, LLC, 2010). Using the estimated Q-score, phylogeny is developed using PHYLIP^25^ with KITSCH algorithm. Pictorial representation of the phylogenetic tree is developed using SPLITSTREE^26^.

#### Sequence conservation analysis

PSI-BLAST^27^ of Wzi protein (UniProt ID: Q8GNN6) is carried out with all the parameters set to default values (Matrix: BLOSUM62, Expect threshold:10, word size:3, Gap: Existence 11 & extension 1 and PSI-BLAST threshold 0.005). Multiple sequence alignment (MSA) of five sequences are performed using COBALT^28^. Similarly, PSI-BLAST is also carried out using the above-mentioned parameters for bacterial vSGLT (PDB ID: 3DH4, UniProt ID: P96169) and the corresponding MSA is generated. Jalview^29^,^30^ is used to edit the generated MSAs.

#### System setup for MD simulations

As the 15 residues of extracellular loop 5 (L5, Fig. 2B) are missing in the crystal structure of Wzi (PDB ID: 2YNK^8^), these residues are modeled using ModLoop^31^,^32^ to obtain the starting model for wild-type Wzi (Wzi-WT) simulations. Three Wzi helix H1 mutants are also generated, namely, H1mut1, H1mut2 and H1mut3 (Table S1) using Wzi-WT as a template. H1mut1 has residues 32–36 of helix H1 mutated to glycine, while H1mut2 and H1mut3 have residues 35–39 and 39–43 of H1 mutated to glycine respectively.

Wzi systems for molecular dynamics simulations are generated using CHARMM-GUI^18^,^33^,^34^,^35^,^36^ web interface. Models are built such that the protein is embedded in the lipid bilayer using replacement method^36^ under heterogeneous membrane builder 36. The orientation of the protein in membrane environment is chosen based on the orientations of proteins in membranes (OPM) database^37^. To mimic the outer membrane of *E. coli*, a heterogenous lipid bilayer is used with POPG and POPE in a 1:3 ratio as discussed elsewhere^38^ by mentioning the number of lipid molecules using CHARMM-GUI interface. The systems are solvated in a rectangular water box using preequlibrated TIP3P water boxes^39^ to a thickness of 17.5 Å on top and bottom of the system, leading to approximately 14,000 water molecules. 150 mM KCl (73 K^+^ ions and 13 Cl^−^) is added to the solvent using Monte-Carlo method^40^. Subsequently, each system is subjected to 0.5 μs simulation that also encompasses 375 ps equilibration run. MD is performed using GROMACS 4.6.4 software^41^ with CHARMM 36^18^,^19^ all atom force field using the following parameters.

MD simulation protocol: During the first step of the equilibration run, each system is subjected to 5000 cycles of steepest descent minimization. This is followed by 50 ps of MD simulation using NVT ensemble using leapfrog algorithm with 1 ps time interval, wherein, Berendsen thermostat^42^ is used to control the simulation temperature with 1 ps time coupling. Subsequently, 25 ps of MD simulation with NPT ensemble with 1 fs time interval is performed followed by 300 ps of MD simulation with 2 fs time step. Berendsen barostat^42^ is used to maintain the semiisotropic pressure (1 bar) with a time coupling 5 ps. Using leapfrog integrator, production run is extened up to 0.5 μs for each system along with Nose-Hoover thermostat^43^ (303 K) and Parrinello-Rahman barostat^44^,^45^ (1bar) with 1 ps and 5 ps coupling respectively. Pressure in the system is maintained in an semiisotropic manner in such a way that pressure in the X-Y plane of the membrane is independent of Z-direction. All the simulations are performed with particle mesh Ewald (PME) method^46^ which is used to treat long-range electrostatic interactions with the cut-off of 1.2 nm. A switching function with the cut-off of 1.0 nm is used to compute the Lenard-Jones potential and the distance up to 1.2 nm is used for the calculation.

50 ns simulations for all the aforementioned systems are also carried out using constant pressure and temperature (CPT dynamics) with CHARMM molecular dynamics program^18^,^19^ using CHARMM 36 all atom force field.

#### Analysis of the trajectories

Quantification of barrel water: To quantify the total number of barrel water molecules per ns, water molecules that fall within the minimum and maximum limits of X & Y coordinates of the entire β-barrel and between −10 Å & 10 Å from the center of the barrel along the Z-axis are summed over every ns.

Quantification of barrel permeation events and diffusion permeability: Barrel permeation is calculated at 5 separate points numbered from 1 to 5 (3 at the extracellular side and 2 at the periplasmic side) (Fig. 3A) as discussed below. When a water molecule that enters into the barrel through anyone of the 3 extracelluar diffusion points and exits the barrel through anyone of the 2 periplasmic diffusion points or vice-a-versa, then, it is counted as an diffusion entry or diffusion exit respectively. For this calculation, water molecules that are residing inside the barrel at any point of time are alone considered.

Diffusion entry calculation: As the tortuous nature of the water diffusion paths complicates diffusion calculation, a method is devised here to define a diffusion plane, through which, a water molecule must pass to be considered for diffusion. A diffusion plane at each point is defined by X, Y & Z coordinates corresponding to the atoms of three specific amino acids (Fig. S8). At first, the center of mass (X_cm_, Y_cm_, Z_cm_) is calculated by considering the atoms corresponding to the X, Y & Z coordinates of three amino acids that are involved in the regulation of water diffusion. Subsequently, a local XY-plane (diffusion plane) is defined with the upper and lower bounds of (X_cm_ + 3.5 Å, Y_cm_ + 3.5 Å) and (X_cm_ − 3.5 Å, Y_cm_ − 3.5 Å) respectively. At the extracellular side, a water entry into the β-barrel (aligned along the Z-axis) is considered only when a water molecule that has Z coordinate greater than Z_cm_ at n^th ^ps subsequently falls within the defined XY-plane with Z-coordinates between Z_cm_ and (Z_cm_ − 3.5 Å) (Z_1_) and soon after it crosses (Z_cm_ − 3.5 Å) (Fig. S8A). Subsequently, diffusion entry is counted only when a water molecule at the periplasmic side that has Z coordinate between Z_cm_ and (Z_cm_ + 3.5 Å) (Z_2_) at n^th^ ps, falls within the defined XY-plane with Z-coordinates between Z_cm_ and (Z_cm_ − 3.5 Å) (Z_1_) and soon after it crosses (Z_cm_ − 3.5 Å) (Fig. S8B).

Diffusion exit calculation: Calculation of periplasmic diffusion is swapped in such a way that a diffusion is considered when a water molecule that has Z-coordinates lesser than Z_cm_ at n^th^ ps, subsequently falls within the defined XY-plane with Z-coordinates between Z_cm_ and (Z_cm_ + 3.5 Å) (Z_2_) and soon after it crosses (Z_cm_ + 3.5 Å) (Fig. S8B). Subsequently, diffusion exit is counted only when a water molecule at the extracellular side that has Z-coordinates between Z_cm_ and (Z_cm_ − 3.5 Å) (Z_1_) at n^th^ ps, falls within the defined XY-plane with Z-coordinates between Z_cm_ and (Z_cm_ + 3.5 Å) and soon after it crosses (Z_cm_ + 3.5 Å) (Fig. S8A). Detailed illustration about the diffusion entry and diffusion exit calculation at each diffusion point is depicted in Fig. S9.

Calculated permeation events are used to estimate the diffusion permeability (p_d_) as described elsewhere^14^ for the last 200 ns of the simulations.

Quantification of barrel water entry and exit: Calculation of barrel water entry and exit is performed by taking into account the water molecules that fall within 5 Å range of center of mass (X_cm_, Y_cm_, Z_cm_) corresponding to each entry point for every 1 ns trajectory. Subsequently, it is checked whether a specific water molecule is between Z_cm_ and (Z_cm_ + 5 Å) or between Z_cm_ and (Z_cm_ − 5 Å). Water entry and exit is calculated by a protocol similar to diffusion entry/exit calculations, but, only covering ±5 Å distance in the Z-dimension. For instance, if the water molecule is between Z_cm_ and (Z_cm_ + 5 Å), it is counted as an extracellular entry soon after it crosses (Z_cm_ − 5 Å) and the reverse is considered for exit. Presence of the water molecule within the XY plane of the appropriate entry point (i.e X_cm_ ± 3.5 & Y_cm_ ± 3.5) during the above calculation is also considered. Similarly, at the periplasmic side, water molecule moving from (Z_cm_ − 5 Å) to (Z_cm_ + 5 Å) is counted as the entry and the reverse is taken into account for exit.

Net flux calculation:  Calculated water entry and exit events are utlized in the net flux estimation. Net flux is calculated by subtracting the total number of water molecules that exit from the barrel through all the entry/exit points from the total number of water molecules that enter inside the barrel through all the entry/exit points over the last 200 ns. It is noteworthy that net flux is calculated by simply considering water entry and exit events from all the entry/exit points, irrespective of whether a water molecule permeates across the channel or not.

Normalized frequency calculation of water entry/exit mechanisms: Following the above-described method, normalized frequency of water entry/exit mechanisms at each point is calculated by additionally imposing a hydrogen bond distance criterion (3.5 Å) between the water oxygen atoms and the side chain/main chain electronegative atoms of the amino acids.

Barrel and helix 1 (H1) interfacial water calculation: To calculate the number of water molecules at the interface of H1 and the barrel, the maximum & minimum values corresponding to X & Z coordinates are calculated by considering the appropriate H1 and barrel residues (Fig. S8C). On the other hand, Y coordinates are chosen with respect to the appropriate barrel residues so as to cover the entire helix H1. A water molecule is counted as interfacial water, only when it falls in the area ascribed by the aforementioned coordinates.

PyMol (The PyMol Molecular Graphics System, Version 1.3, Schrodinger, LLC, 2010) is used for the visualization of all the simulated trajectories and VMD^47^ is used for the generation of movies. All the graphs are plotted using MATLAB (MATLAB 7.11.0, The MathWorks Inc., Natick, MA, 2010).

## Additional Information

**How to cite this article**: Sachdeva, S. *et al*. Key diffusion mechanisms involved in regulating bidirectional water permeation across *E. coli* outer membrane lectin. *Sci. Rep.* **6**, 28157; doi: 10.1038/srep28157 (2016).

## Supplementary Material

Supplementary Information

Supplementary Movie S1

Supplementary Movie S2

Supplementary Movie S3

Supplementary Movie S4
