# Supplementary material for: Key diffusion mechanisms involved in regulating bidirectional water permeation across E. coli outer membrane lectin
Source: Sci Rep. 2016 Jun 20;6:28157. doi: 10.1038/srep28157 (PMC4913347; doi:10.1038/srep28157)
Supplement: Supplementary Information [file srep28157-s1.doc]

**Title:**

Key diffusion mechanisms involved in regulating bidirectional water permeation across *E. coli* outer membrane lectin

Shivangi Sachdeva^#^, Narendar Kolimi^#^, Sanjana Anilkumar Nair^#^ and Thenmalarchelvi Rathinavelan^*^

Department of Biotechnology, Indian Institute of Technology Hyderabad, Kandi,

Telangana State 502285, India

Phone: 91 40 2301 7067, Fax: 91 40 2301 6032

^#^Equal contribution

*For Correspondence: tr@iith.ac.in

**Running title:**

*E. coli* outer membrane lectin is also a bidirectional water selective porin

**Keywords:** Group 1 capsular polysaccharide, *E. coli* outer membrane lectin, Wzi, K30_CPS_, Molecular dynamics simulation, YQF motif, β-barreled water specific porin, transient water wires, membrane insertion, osmoregulation, diffusion permeability

**Movie 1:** Filling up the β-barrel of Wzi (cyan colored cartoon) with water molecules (red & white colored sphere).

**Movie 2:** Extracellular to periplasmic side water (red & white colored sphere) permeation event across Wzi (orange colored cartoon). Water entry & exit points are shown in stick representation and the hydrophobic plug is shown in yellow color spheres.

**Movie 3:** Periplasmic side to extracellular to water (red & white colored sphere) permeation event across Wzi (orange colored cartoon). Water entry & exit points are shown in stick representation and the hydrophobic plug is shown in yellow color spheres.

**Movie 4:** Splaying and insertion of L5 residues (colored yellow) into the membrane bilayer. Note that the protein and membrane are shown in orange colored cartoon and green colored surface respectively.

| 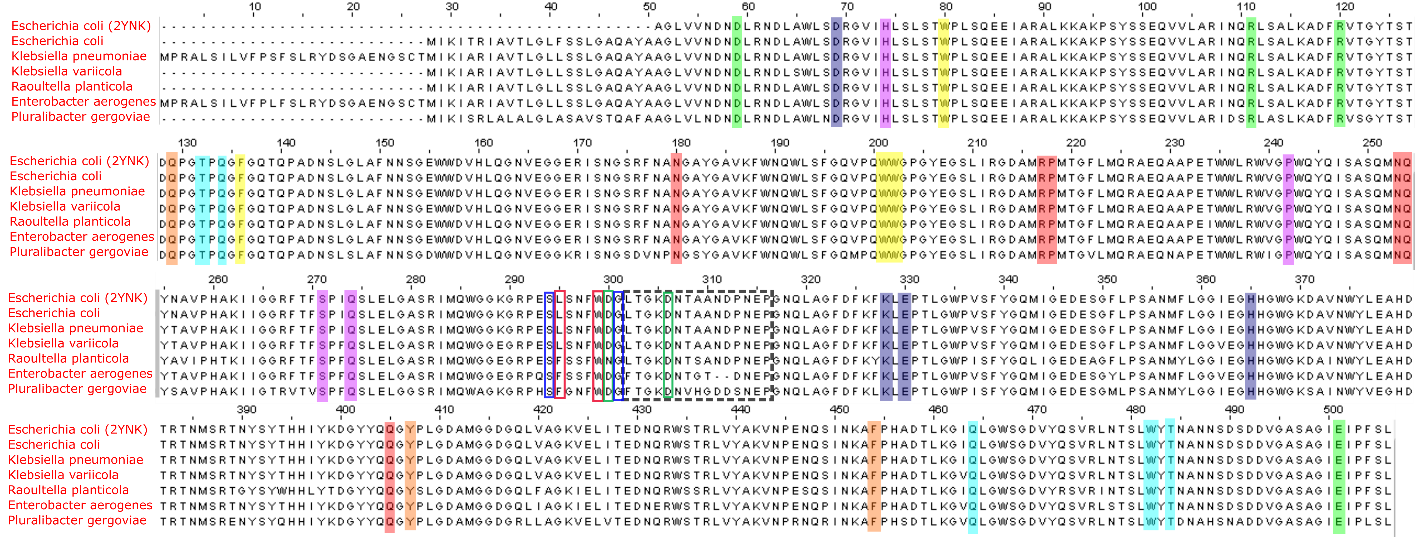 |
| --- |
| **Figure S1**. Multiple sequence alignment of *E. coli* outer membrane lectin (Wzi) with Wzi from *Klebsiella pneumoniae, Klebsiella variicola, Raoultella planticola, Enterobacter aerogenes* and *Pluralibacter gergoviae.* Conserved residues relevant to the current investigation have been highlighted (Figures 4-8): Five diffusion points are depicted in orange (point 1: Y380,Q102&F427), red (point 2: N153,R190,P191,N226,Q227&Q378) and cyan (point 3: T105,Q107,Q437,W455&T457), green (point 4a&4b: D32,R84,R93&E474), blue (point 5a: D42,K310,E303&H338) and purple (point 5b: H47,P215,S244&E247) filled boxes. Conserved hydrophobic plug (W53,F109,W174&W175) is shown in filled yellow box. L5 residues involved in hydrophobic (L268&W272), electrostatic (D273&D279) & hydrogen bonding (S267&G274) interactions with the lipid membrane are shown in red, green and blue transparent boxes respectively. L5 residues that are absent in the crystal structure (L275-T276-G277-K278-D279-N280-T281-A282-A283-N284-D285-P286-N287-E288-P289) are shown in black transparent dotted box. |

| 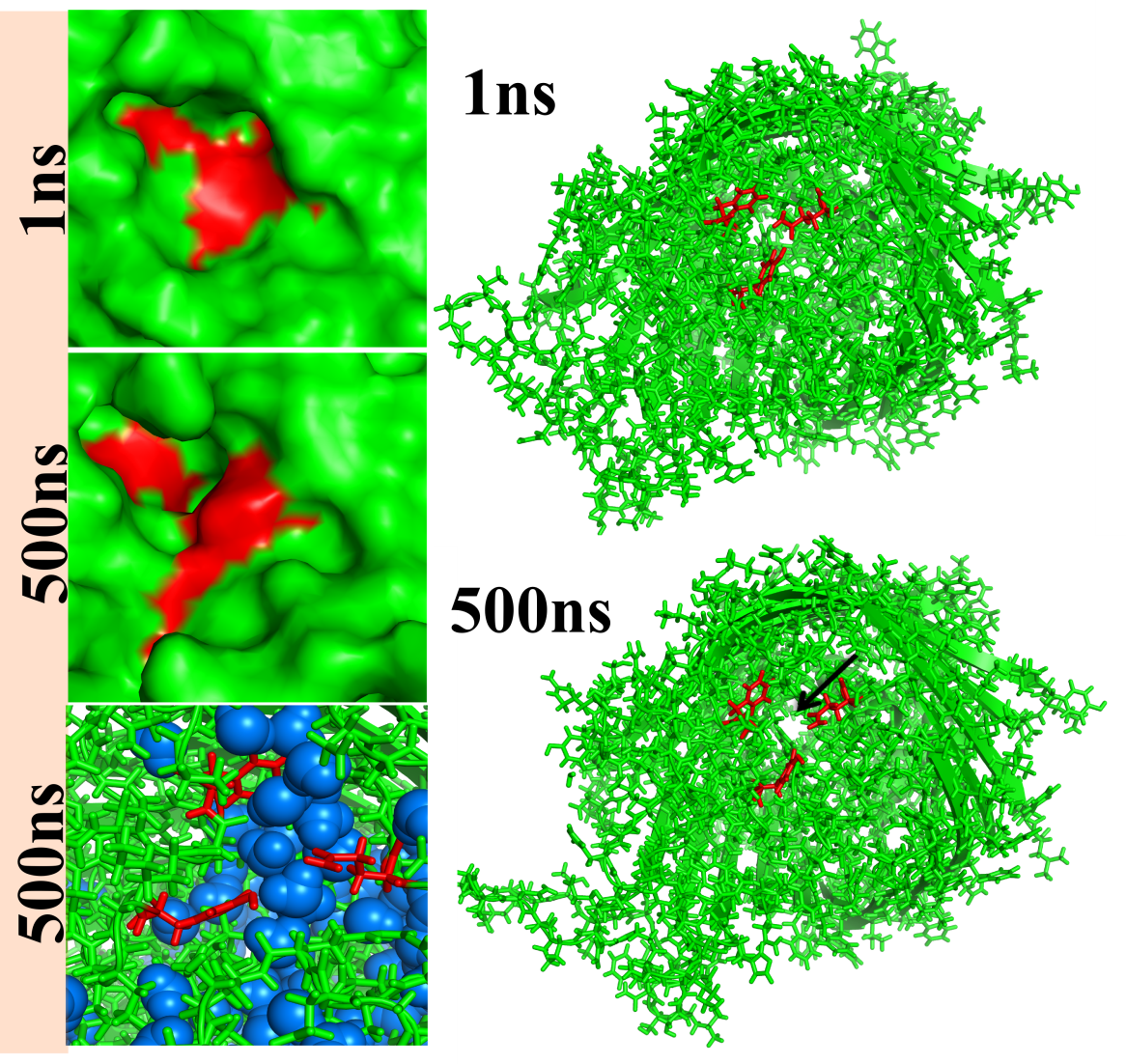 |
| --- |
| **Figure S2. Extracellular pore opening at YQF triad of Wzi.** (Left) Snapshots of extracellular face of Wzi (green surface) at 1ns (Top-Left) and 500ns(Middle-Left & Bottom-Left) illustrating the pore opening at YQF triad (colored red). Note that the open state structure at 500ns (representative structure for open state) facilitates water diffusion (blue spheres, Bottom-Left) through the YQF triad (shown in red sticks) as compared to the closed state structure at 1ns (Top-Left). (Right) Stick representation of Figures given in Top-Left (1ns) & Middle-Left(500ns) with YQF triad colored red. Black arrow indicates opened pore (Bottom-right). |

| 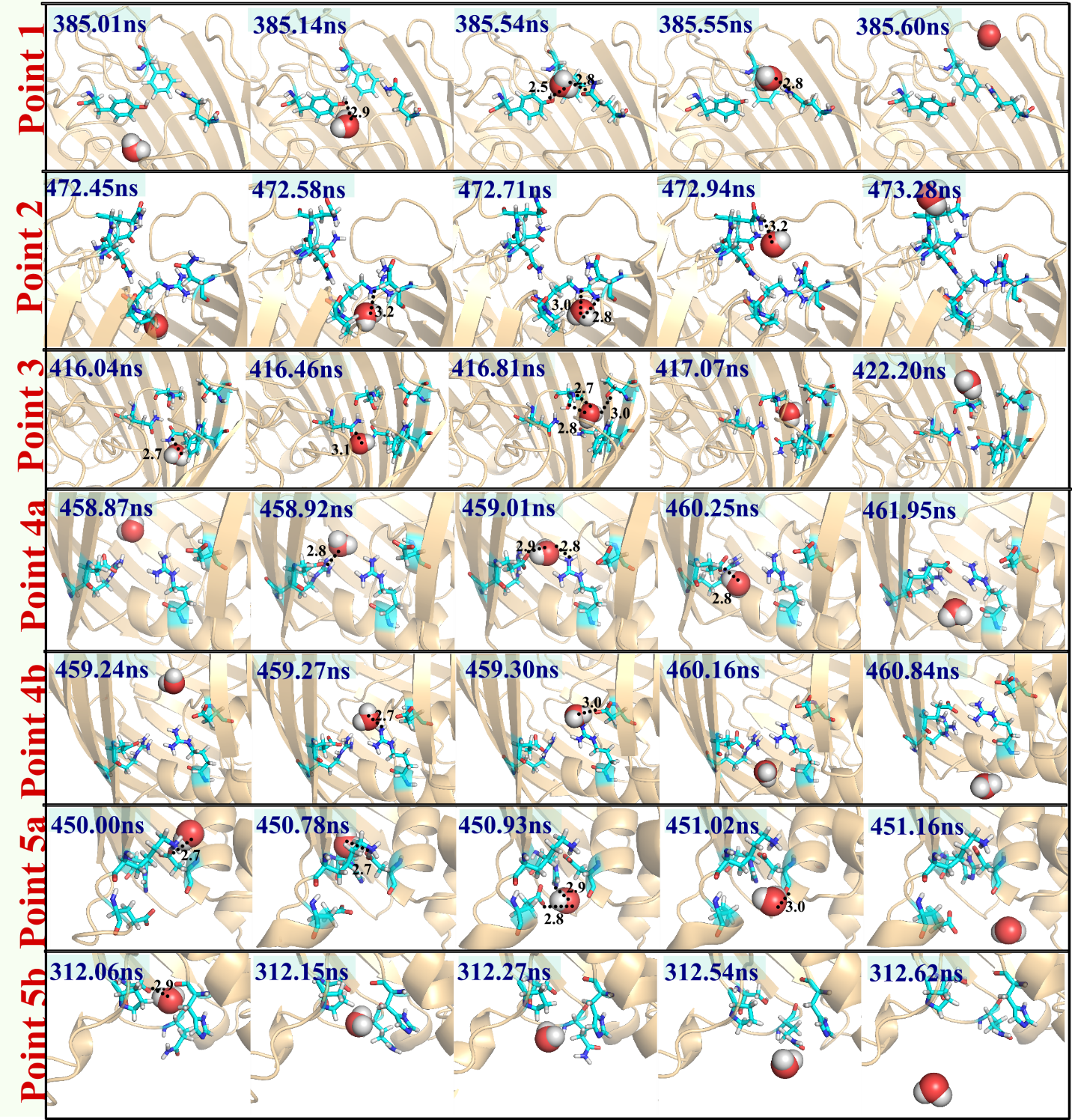 |
| --- |
| **Figure S3. Wzi water exit mechanisms.** Snapshots illustrating the water exit at each of the five barrel diffusion points (1 to 5) with the corresponding time marked. Water molecule is shown as spheres and the residues responsible for mediating the diffusion at each of the five points are depicted as sticks. |

| 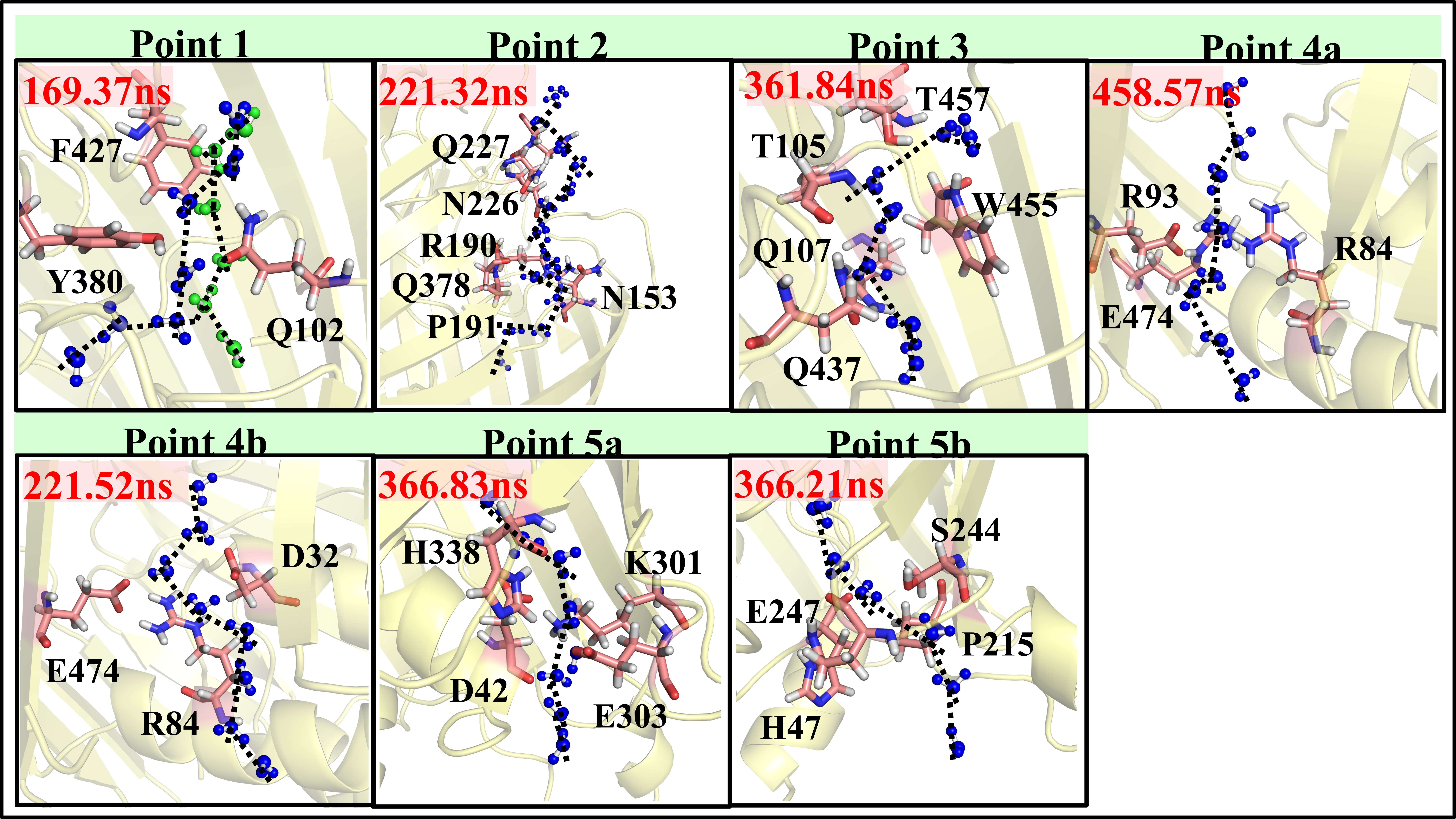 |
| --- |
| **Figure S4. Transient water wires formed at the diffusion points.** Snapshots depicting transient water wires involved in water entry/exit at all the five diffusion points (marked as Point 1, 2, 3, 4a, 4b and 5a & 5b) with the corresponding time indicated. Water oxygens within 3.5Å distances are connected by dotted lines to illustrate the formation of water wires. Water molecules participating in the water wire formation are represented as blue colored ball and stick unless otherwise mentioned. Note the formation of 2 transient water wires at the YQF diffusion point (point 1) in contrast to a single water wire in the other points. At point 1, water wire between unlocked Y380…Q102 residues is shown in blue and the one at the interspace of F427&Y380…Q102 is shown in green. |

| 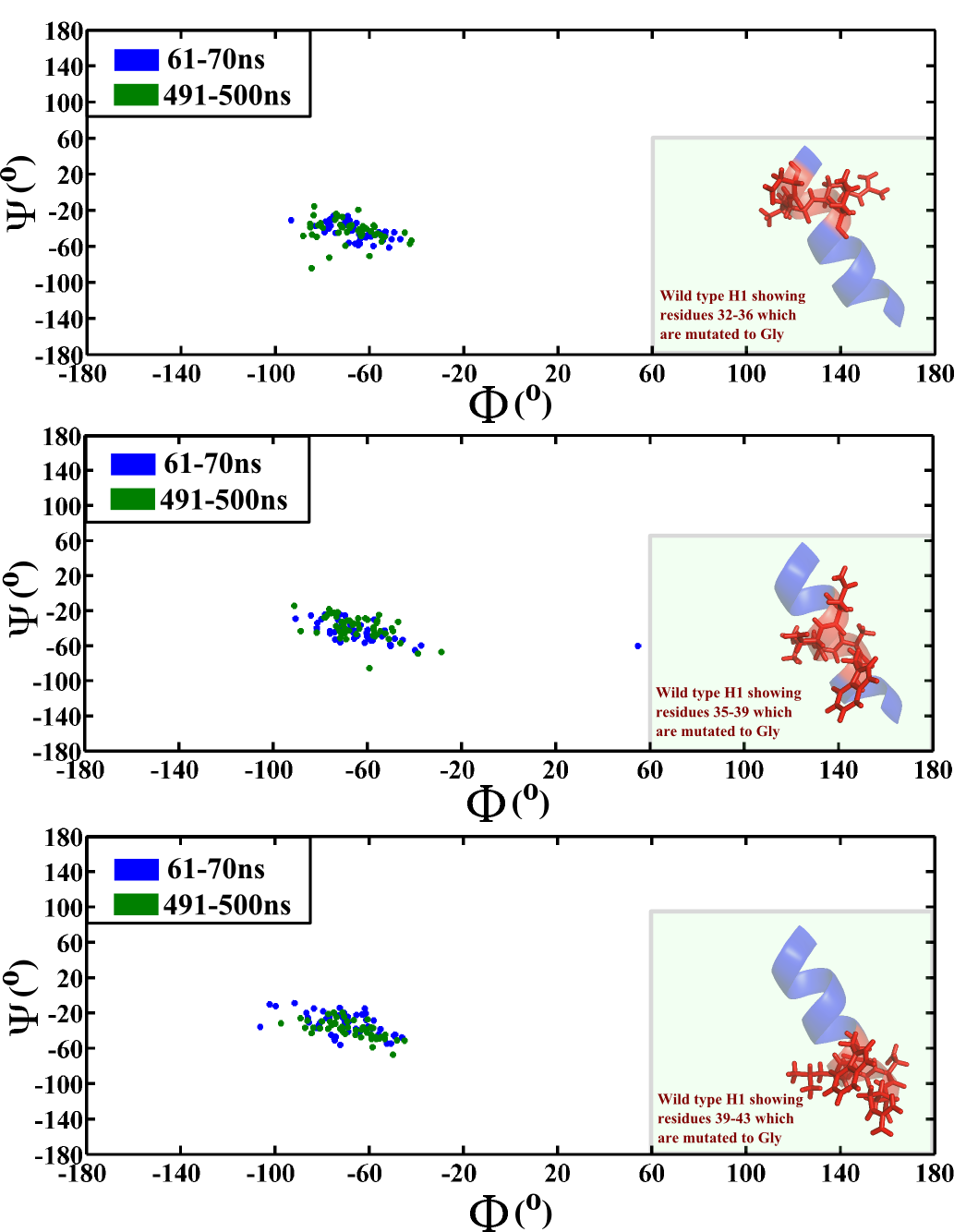 |
| --- |
| **Figure S5.** Ramachandran diagram of pentaglycine mutants H1mut1 (Top), H1mut2 (Middle) and H1mut3 (Bottom) calculated over the first 10ns of the equilibrium state (blue) and last (green) 10ns of the simulations. (Inset) Mutation sites corresponding to H1mut1, H1mut2 and H1mut3 are mapped onto Wzi-WT helix H1 (cartoon representation) in red sticks. |

| 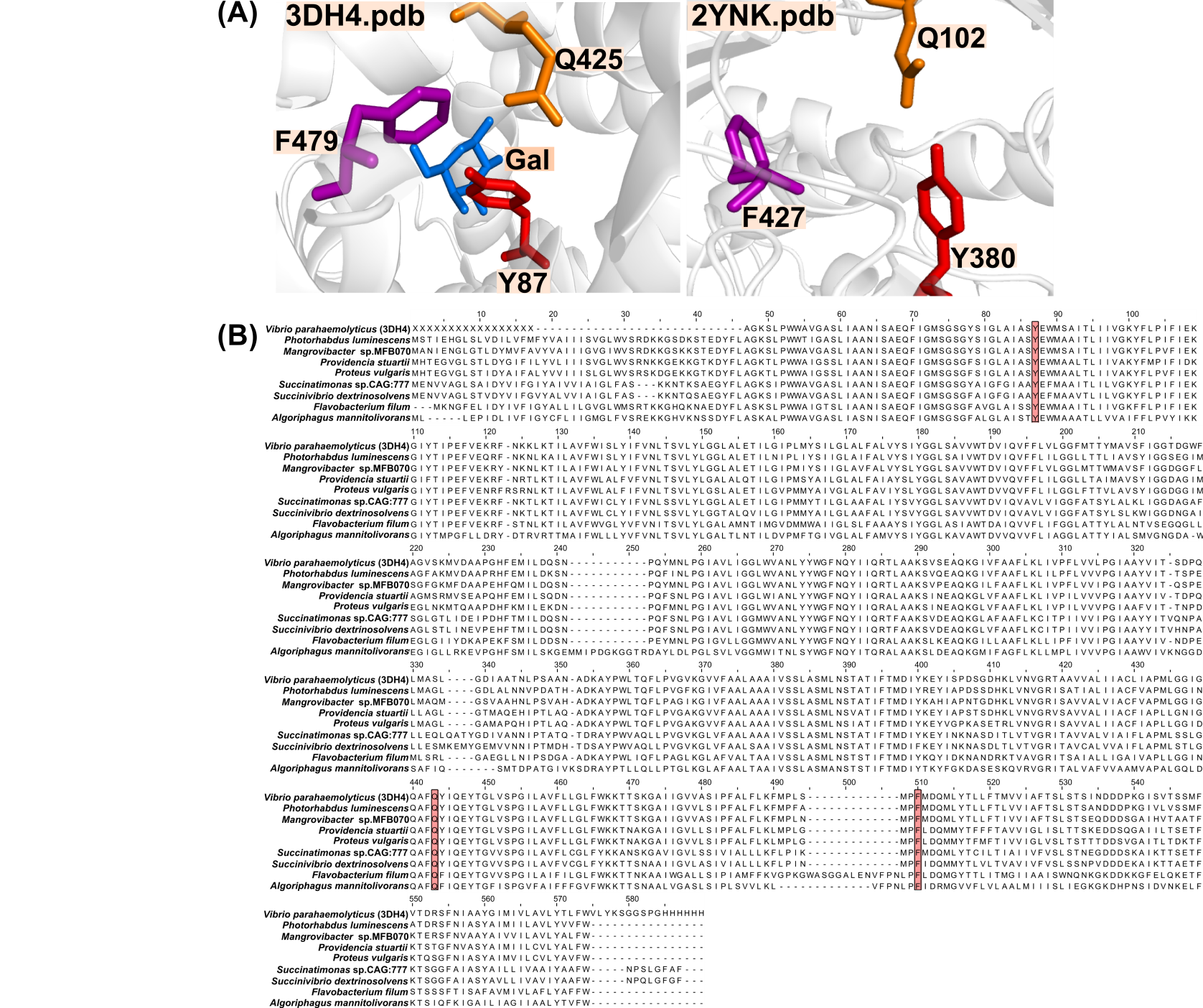 |
| --- |
| **Figure S6. Similarity in sugar binding site of *Vibrio parahaemolyticus* sodium-galactose cotransporter (vSGLT)(PDB ID: 3DH4) and ‘YQF’ triad of Wzi (PDB ID: 2YNK).** (A) Zoomed view of galactose (blue stick) binding site of vSGLT and ‘YQF’ triad of Wzi with Y (red), Q (orange) and F (purple) represented in stick. (B) Multiple sequence alignment of vSGLT (SGLT in *Vibrio parahaemolyticus)* with the sugar transporters of *Photorhabdus luminescens, Mangrovibacter*sp.MFB070*, Providenciastuartii, Proteus vulgaris, Succinatimonas*sp.CAG:777*, Succinivibrio dextrinosolvens, Flavobacterium filum* and *Algoriphagus mannitolivorans.* Note that conserved ‘YFQ’ triad is highlighted in saffron. |

| 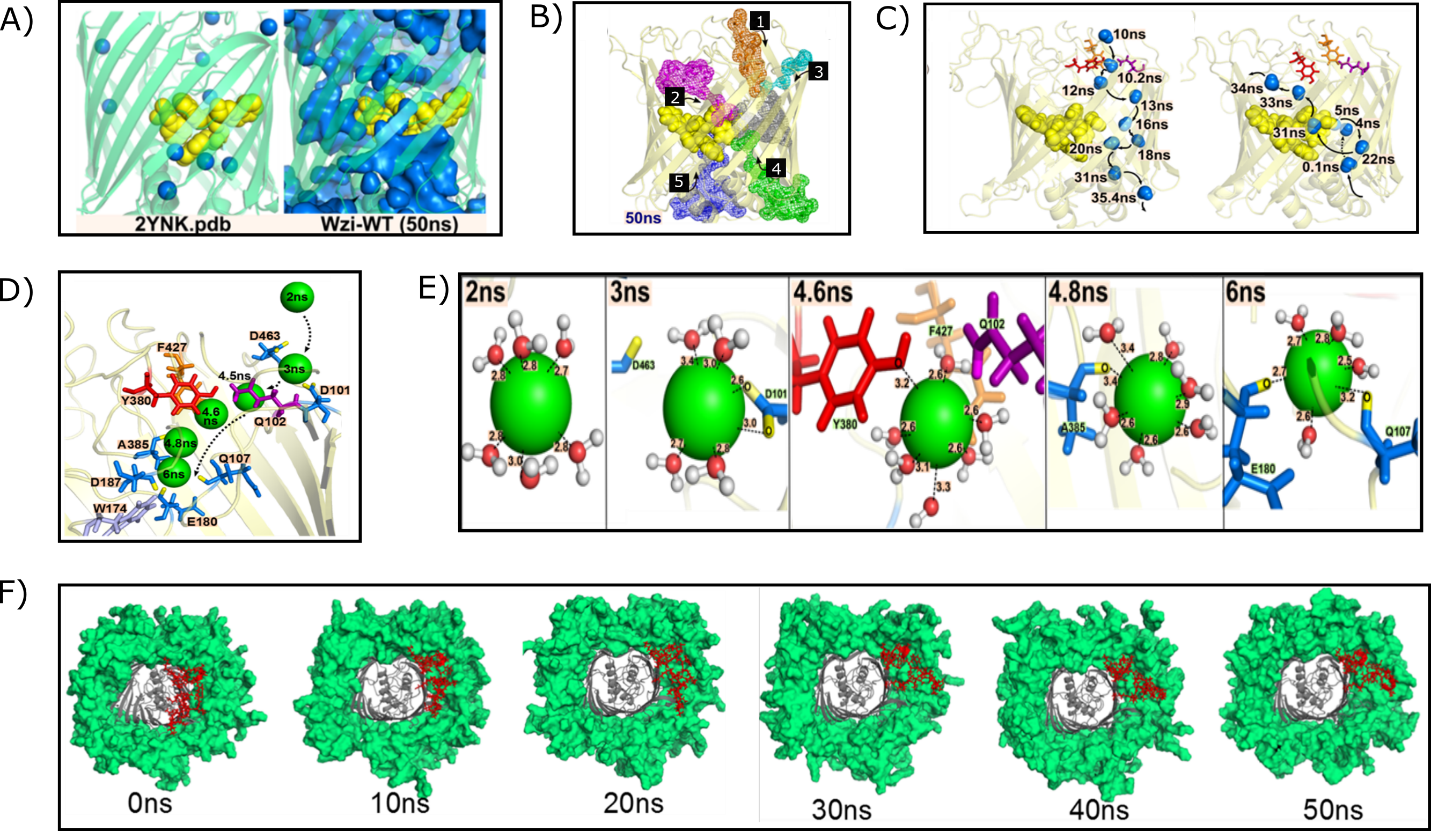 |
| --- |
| **Figure S7. Summary of CHARMM simulation**. 50ns preliminary MD simulation carried out using CHARMM molecular modeling program also confirms about: (A) porin like characteristics of Wzi (B) involving 5 different entry/exit points. (C) Complete crossing event from either directions (extracellular to periplasmic (Left) and vice-a-versa (Right)), (D&E) ion binding pocket and (F) role of loop 5 in membrane insertion are also observed. |

| 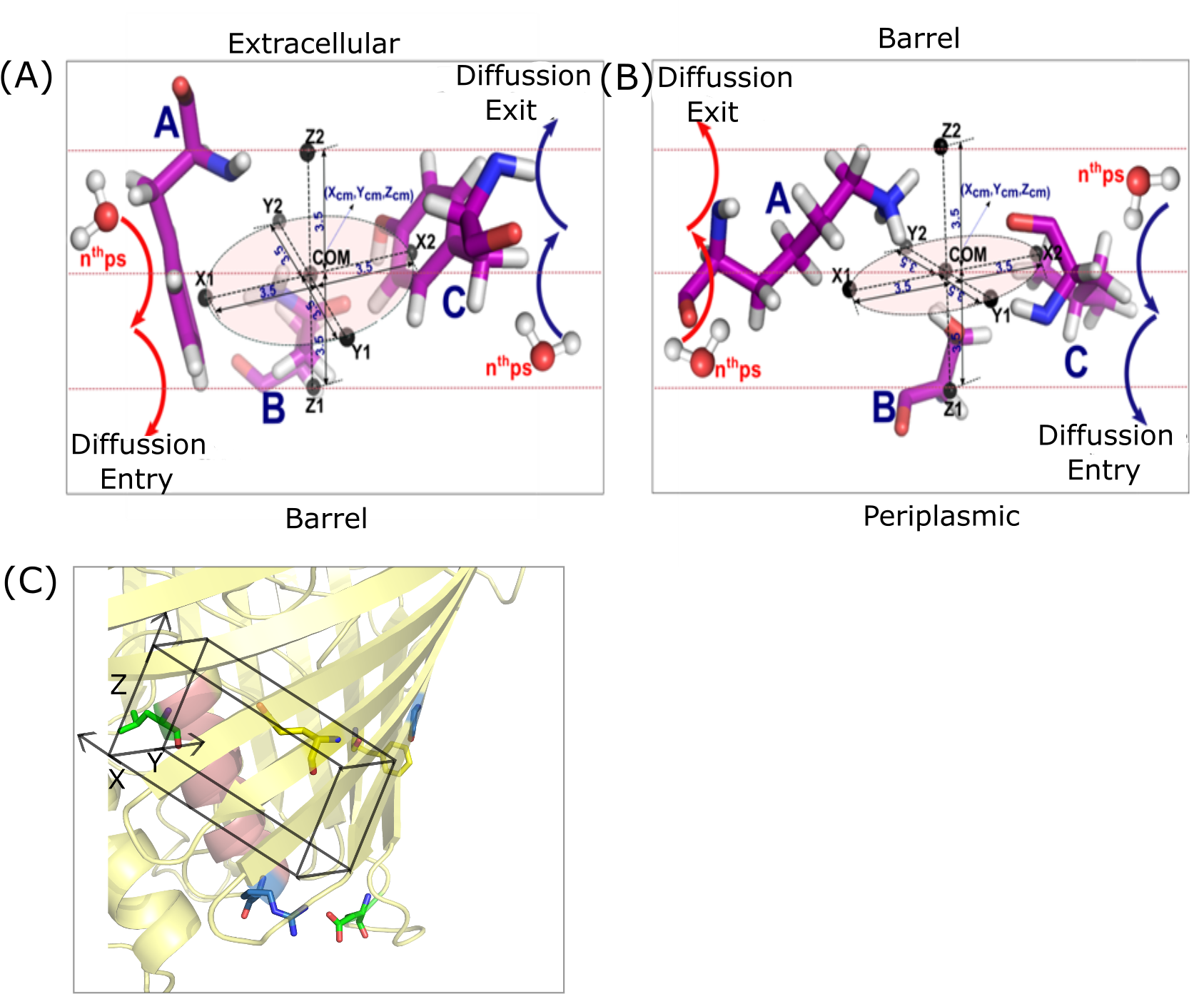 |
| --- |
| **Figure S8. (A&B) Definition of water diffusion plane.** Diffusion plane defined by the side chains of three amino acids A,B&C (given in sticks) at the (**A**) extracellular and (**B**) periplasmic sides is represented in a transparent ellipse. A water molecule, using which, the barrel entry (colored red) and exit (colored blue) calculations are explained in Methods section is shown in ball and stick representation. Note that (X_cm_,Y_cm_,Z_cm_) correspond to the center of mass (COM) calculated with respect to the atoms of the 3 amino acids and it falls at the center of the plane. (X_1_,Y_1_,Z_1_) & (X_2_,Y_2_,Z_1_) that are displaced to a distance of +3.5Å and -3.5Å from (X_cm_,Y_cm_, Z_cm_) respectively are connected through dotted lines. **(C) Pictorial representation illustrating the interfacial water calculation.** See Methods for details. |
| 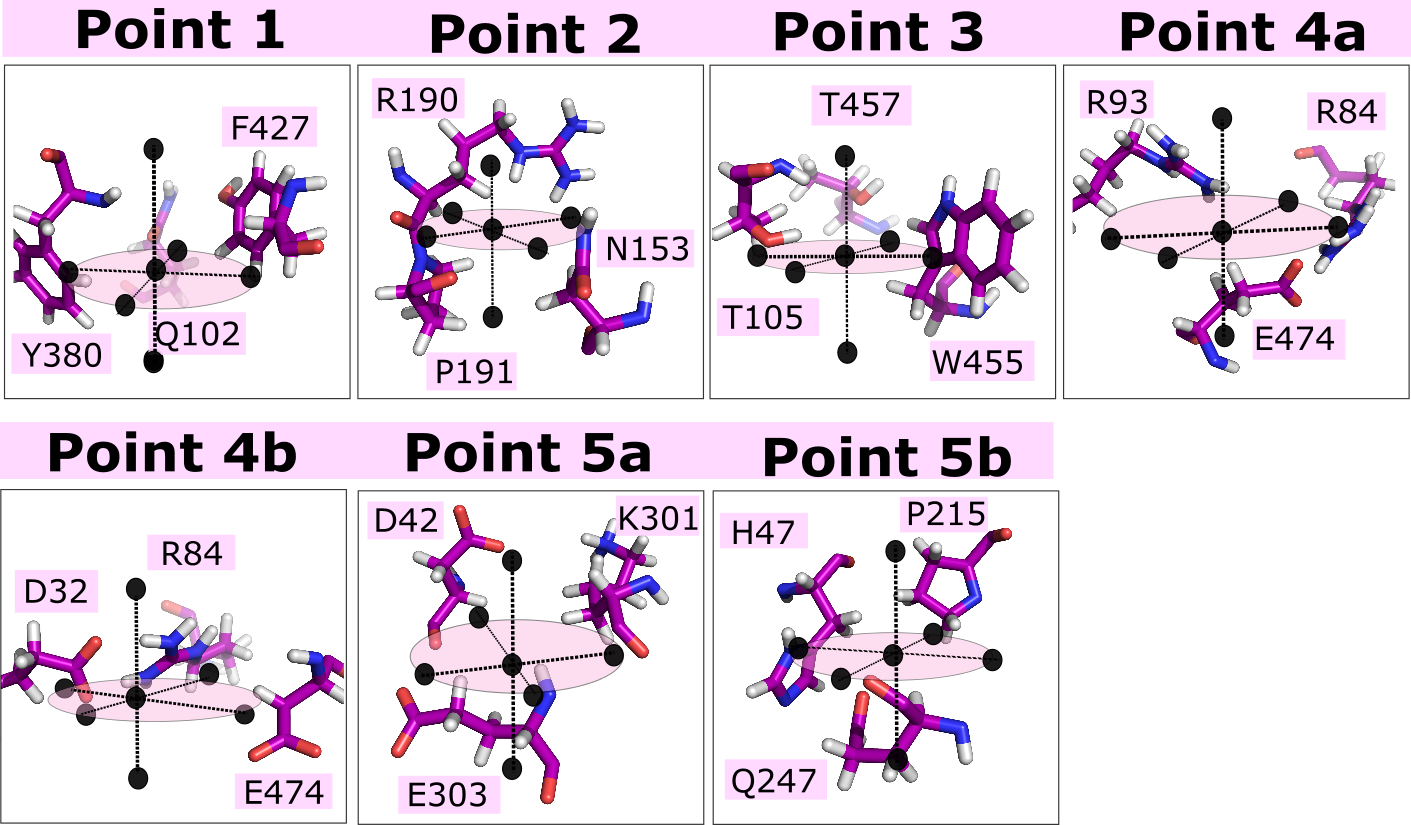 |
| **Figure S9. Pictorial representation of residues used in the calculation of barrel water entry/exit through diffusion points 1-5.** Three amino acids that are considered for the calculation of barrel entry/exit are shown in stick representation with the (X_cm_,Y_cm_,Z_cm_), (X1,Y1,Z1) & (X2,Y2,Z2) shown in black spheres and reference plane is marked in transparent ellipse. Note that (X_cm_,Y_cm_,Z_cm_) corresponds to the center of mass calculated with respect to the atoms of the three amino acids and it falls at the center of the plane. (X1,Y1,Z1) & (X2,Y2,Z2) are connected to (X_cm_,Y_cm_,Z_cm_) through dotted lines. For details, refer Methods and Figure 12 in the main text. |

| **Table S1. Description of systems considered for 500ns simulations** | |
| --- | --- |
| **System** | **Description** |
| Wzi-WT | Wziwild type structure (with modeled extracellular loop 5 (L5) residues: **L_275_TGKDNTAANDPNEP_289_**) |
| H1mut1 | Residues 32-36 of helix H1 mutated to Glycine |
| H1mut2 | Residues 35-39 of helix H1 mutated to Glycine |
| H1mut3 | Residues 39-43 of helix H1 mutated to Glycine |
